# Multidimensional Observations of Dissolution-Driven Convection in Simple Porous Media Using X-ray CT Scanning

**DOI:** 10.1007/s11242-018-1158-3

**Published:** 2018-10-01

**Authors:** Rebecca Liyanage, Jiajun Cen, Samuel Krevor, John P. Crawshaw, Ronny Pini

**Affiliations:** 1 Department of Chemical Engineering, Imperial College London, London, UK; 2 Qatar Carbonates and Carbon Storage Research Centre, Imperial College London, London, UK; 3 Department of Earth Science and Engineering, Imperial College London, London, UK

**Keywords:** Solute mixing, 3D imaging, Porous media

## Abstract

We present an experimental study of dissolution-driven convection in a three-dimensional porous medium formed from a dense random packing of glass beads. Measurements are conducted using the model fluid system MEG/water in the regime of Rayleigh numbers, $Ra=2000{-}5000$. X-ray computed tomography is applied to image the spatial and temporal evolution of the solute plume non-invasively. The tomograms are used to compute macroscopic quantities including the rate of dissolution and horizontally averaged concentration profiles, and enable the visualisation of the flow patterns that arise upon mixing at a spatial resolution of about ($2\times 2\times 2)\,\hbox {mm}^3$. The latter highlights that under this *Ra* regime convection becomes truly three-dimensional with the emergence of characteristic patterns that closely resemble the dynamical flow structures produced by high-resolution numerical simulations reported in the literature. We observe that the mixing process evolves systematically through three stages, starting from pure diffusion, followed by convection-dominated and shutdown. A modified diffusion equation is applied to model the convective process with an onset time of convection that compares favourably with the literature data and an effective diffusion coefficient that is almost two orders of magnitude larger than the molecular diffusivity of the solute. The comparison of the experimental observations of convective mixing against their numerical counterparts of the purely diffusive scenario enables the estimation of a non-dimensional convective mass flux in terms of the Sherwood number, $Sh=0.025Ra$. We observe that the latter scales linearly with *Ra*, in agreement with both experimental and numerical studies on thermal convection over the same *Ra* regime.

## Introduction

The study of convective mixing in porous media continues to find applications in both traditional and emerging engineering problems, many of which occur in natural environments (Gebhart and Pera 1971; Diersch and Kolditz 2002). We focus here on density-driven *free* convection to highlight that the mixing process is induced and sustained by a buoyancy effect, in the absence of advective flows that are introduced by, e.g. an external pressure gradient. One particular application that has increased the interest in this phenomenon is geologic carbon sequestration (GCS) (Huppert and Neufeld 2014), because of its potential impact on the dissolution rate of $\hbox {CO}_2$ into formation fluids. In this scenario, the buoyancy effect may be due to the natural occurring geothermal gradient in the reservoir, but also -and primarily- to varying composition of the aqueous phases (Lindeberg and Wessel-Berg 1997). In fact, $\hbox {CO}_2$, dissolution into brine leads to a local density increases on the order of 0.1–1% [depending on pressure and temperature (Efika et al. 2016)], which is sufficient to create a buoyant instability that in turn induces a convective overturn in the brine; the denser $\hbox {CO}_2$-rich aqueous mixture flows downwards and pushes fresh brine up towards the $\hbox {CO}_2$-brine interface. The ability of $\hbox {CO}_2$-saturated brine to sink deeper into the aquifer reduces the likelihood of $\hbox {CO}_2$ leakage, thereby increasing long-term storage security. Dissolution of $\hbox {CO}_2$ is considered a key trapping mechanism in GCS (Benson and Cole 2008) and convective mixing is expected to contribute largely to this process (Ennis-King and Paterson 2005), partly because mass transfer by diffusion, despite being ubiquitous, is very slow. Recent surveys of potential storage sites around the world suggest that the conditions are often met for convective mixing to occur [e.g. Sathaye et al. (2014) using data compiled in Szulczewski et al. (2012)]; however, estimates on its actual contribution towards storage, its spatial footprint and its timescale are still largely uncertain, because of the lack of direct observations at representative subsurface conditions and the intrinsic difficulty in estimating dimensions and properties in heterogeneous rock formations (Sathaye et al. 2014).

There have been numerous experimental studies where density-driven convection has been investigated in the context of GCS. These efforts may be broadly divided in two categories, namely (i) studies using high-pressure blind PVT cells and (ii) those using 2D transparent Hele-Shaw cells. The former can be operated with representative fluids (e.g. supercritical $\hbox {CO}_2$ and brine) and the rate of dissolution is typically inferred from pressure decay (Yang and Gu 2006; Farajzadeh et al. 2009; Khosrokhavar et al. 2014) and/or changes in weight (Arendt et al. 2004) inside the closed reactor, or is measured directly by recording the make up liquid volume needed to maintain a constant pressure in the system (Newell et al. 2018). For data reconciliation, some authors have applied the diffusion equation with an effective diffusion coefficient (Yang and Gu 2006; Moghaddam et al. 2012), while others have used more rigorous mathematical models that account for both mass and momentum conservation in the liquid phase (and that use the bulk molecular diffusivity) (Khosrokhavar et al. 2014; Farajzadeh et al. 2009). Results from these studies consistently show that under the convective regime the mass-transfer rate across the $\hbox {CO}_2$/brine interface is indeed much faster than that predicted by Fickian diffusion (with an effective diffusion coefficient that is one to two orders of magnitude larger than the (bulk) molecular diffusivity, depending on the initial gas pressure and salt concentration in the brine). Unfortunately, the majority of these observations still refer to the dissolution of $\hbox {CO}_2$ into bulk brine and experiments using porous media have just begun (Newell et al. 2018; Nazari Moghaddam et al. 2015). Also, only in rare cases did the experiments enable direct visualisation of convective patterns (through an embedded optical side cell) (Khosrokhavar et al. 2014; Arendt et al. 2004).

With the intention of visualising the convective process, several authors have made use of Hele-Shaw cells, albeit with analogue fluid-pairs [e.g. MEG-water (Neufeld et al. 2010), water-PG (Backhaus et al. 2011; Tsai et al. 2013; Macminn and Juanes 2013; Agartan et al. 2015), gaseous $\hbox {CO}_2$-water (Kneafsey and Pruess 2010) and $\hbox {KMnO}_4$ in water (Slim et al. 2013; Ching et al. 2017). By enabling direct access to local measures of convection (e.g. wavelength of the instability, vertical plume velocity, plume width and their statistics) (Slim et al. 2013; Ecke and Backhaus 2016), these experiments have been pivotal in supporting the significant effort that has been dedicated to the study of density-driven convection in porous media by means of numerical simulations [see the recent review Emami-Meybodi et al. (2015) and references therein]. Evidence (Hidalgo et al. 2012; Raad and Hassanzadeh 2015; Jafari Raad et al. 2016) now exists that results using analogue fluid pairs may not be directly applicable to the subsurface $\hbox {CO}_2$/brine system (see also Sect. 5); these studies have also demonstrated that despite its inherent chaotic nature, the process of convective mixing can be parameterised in terms of useful macroscopic variables, such as the Rayleigh, *Ra*, and Sherwood number, *Sh* (or its counterpart for heat transfer studies, the Nusselt number, *Nu*). Nevertheless, because the convection process in a porous medium is three-dimensional, concerns have been raised with regard to the inherent limitations of two-dimensional experiments (or of their numerical counterparts) (Lister 1990) and to the applicability of the obtained scaling laws. On the one hand, some authors have proposed that for $Ra>2000$, a scaling relationship exists for the convective mixing process that is universal, both in two and three dimensions (Fu et al. 2013). On the other hand, results from numerical simulations suggest that in three dimensions (i) the dissolution flux is 25–40% larger than in two dimensions (Pau et al. 2010; Hewitt et al. 2014), (ii) stronger dispersion occurs (thus leading to weaker flow) and (iii) fingers grow bigger (thus leading to faster penetration) (Knorr et al. 2016). Experimental validation of these findings is still lacking.

Because of the inherent difficulty of imaging the convective process within an opaque medium non-invasively, very limited experimental observations exist of density-driven convection in three-dimensional porous media. In their early seminal work, Bories and Thirriot (1969) used photographs of the top free surface of a liquid-saturated ($70\times 50\times 8)\,\hbox {cm}^3$ rectangular beadpack to infer fluid movements within the medium itself; most significantly, they demonstrated that cellular structures appear with length scale $\mathcal {O}(l)\sim 10\,\hbox {cm}$, which are not possible in two-dimensional settings, as the latter limit the growth of the plume to two orthogonal directions. These findings were later confirmed by Lister (1990), who used a similar experimental approach and extended these observations to the regime $\mathcal {O}(Ra)\sim 1000$. The first images of the convection pattern *within* a porous medium were reported only a few years later by Howle et al. (1993, 1997) using a shadowgraphic techniques and by Shattuck et al. (1995) using magnetic resonance imaging (MRI) for both regular and disordered packings. In these experiments, observations were limited to $\mathcal {O}(Ra)\sim 100$ and two two dimensions (horizontal flow patterns), and the mixing process was driven by temperature gradients, rather than dissolution. Nevertheless, by demonstrating a novel ability to image the convective process within opaque media non-invasively, these studies have provided direct evidence that the structure of the medium plays a fundamentally important role in the determination of the flow pattern.

In this study, we build on the findings above by presenting multidimensional observations of convective dissolution in simple porous media using X-ray computed tomography (X-ray CT) for the MEG-brine fluid pair. Together with the recent work by Nakanishi et al. (2016) and Wang et al. (2016), we provide what are, to our knowledge, the first non-invasive determinations of three-dimensional patterns in opaque, random porous media. Experiments are carried out in the regime $\mathcal {O}(Ra)\sim 1000$ and the mixing process is quantified using various metrics, including the rate of dissolution and effective diffusion coefficients. Observations are compared to the limiting case of a purely diffusive scenario, which further enables the investigation of a $Sh{-}Ra$ scaling law and its comparison with results reported in the literature using a similar fluid pair.

## Experimental

### Porous Medium and Fluids

**Fig. 1:**
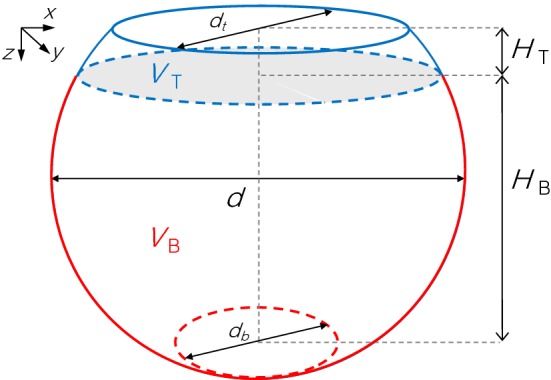
Drawing of the experimental geometry used for the convective dissolution experiments. The bowl is packed with soda glass ballotini ($d_\mathrm{p}\approx 0.5\,\hbox {mm}$); the top and bottom sections of the bowl ($H_\mathrm{B}/H_\mathrm{T}\approx 5.5$) are initially saturated with MEG and brine solutions, respectively. Other dimensions are: $d=18\,\hbox {cm}$, $d_\mathrm{t}=11\,\hbox {cm}$ and $d_\mathrm{b}=8.5\,\hbox {cm}$

The experiments have been conducted in a 3 L acrylic plastic bowl packed with soda glass ballotini ($d_\mathrm{p}=0.5\,\hbox {mm}$, SiLibeads$^{\circledR }$, supplied by VWR, UK). This spherical geometry was selected to reduce imaging artefacts associated with the acquisition of X-ray tomograms of objects with straight edges. The bowl is depicted in Fig. 1; it has dimensions 18 cm $\times$ 15 cm ($d\times H$, where $H=H_\mathrm{T}+H_\mathrm{B}$) and an opening diameter $d_\mathrm{t}=11\,\hbox {cm}$. The porosity of the packing is $\phi \approx 0.36$ and its permeability is estimated from the Kozeny–Carman equation, i.e. $k=\phi ^3d_\mathrm{p,50}^2/(150(1-\phi )^2)=1.9\times 10^{-10}\,\hbox {m}^2$.

**Fig. 2:**
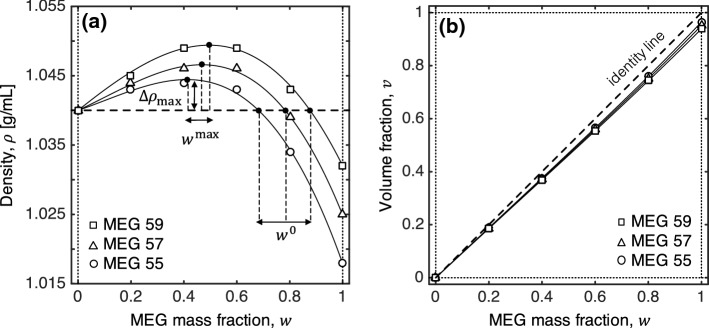
**a** Density curves of the three solution-pairs used in this study, namely MEG55, MEG57 and MEG59 (solution 1 with mass fraction *w*) mixed with brine (solution 2 with mass fraction, $1-w$). **b** Volume fraction of MEG in solution, *v*, as a function of its mass fraction, *w*. In both plots, symbols are experimental results, while the curves represent fitted polynomials of the form, $\rho =a_0+a_1w+a_2w^2+a_3w^3$. Characteristic points on each curve are the maximum density difference achieved upon mixing ($\Delta \rho _\mathrm{max}$), the corresponding weight fraction of the solution ($w^\mathrm{max}$) and the point of neutral buoyancy, $w^0$). The values of these parameters are given in Table 1

The working fluids used in this study are solutions of methanol and ethylene-glycol (MEG, fluid 1) and brine (fluid 2). In particular, three mixtures of ethylene-glycol and methanol (both anhydrous, 99.8%, Sigma-Aldrich) were prepared that differ in wt% ethylene-glycol, namely 55 wt% (MEG55), 57 wt% (MEG57) and 59 wt% (MEG59). The obtained solutions are subsequently doped with 9 wt% potassium iodide (KI, ReagentPlus^®^, 99%, Sigma Aldrich) to achieve high X-ray imaging contrast for the experiments. Only one brine solution is used that contains 6 wt% sodium chloride (NaCl, $>99$%, Sigma Aldrich) in distilled water. The density of the pure solutions and of their mixtures have been measured using an oscillating U-tube density meter (DM5000 by Anton Paar) at 20$^\circ$C and 1 atm. For each measurement, approximately 3 mL of solution was used and the density was taken to be the average of three repeated measurements. The obtained density curves are shown in Fig. 2a as a function of wt% MEG, *w*, where the experimental values (symbols) are plotted alongside fitted polynomial curves (parameters provided in “Appendix A1”). Error bars are not shown in the figure, because they are smaller than the symbols. These curves present a characteristic non-monotonic profile with a maximum at intermediate MEG concentrations ($w=0.4-0.5$) and a density larger than that of pure brine ($\rho (w)>\rho _2$), whereas at larger concentrations ($w>0.7$) the solution becomes buoyant ($\rho _1<\rho (w)<\rho _2$). The key characteristic properties of the solutions are summarised in Table 1, together with estimates of the Rayleigh number, *Ra*. The latter is calculated as:

1 $$\begin{aligned} Ra=\frac{k\Delta \rho _\mathrm{max}H_\mathrm{B}g}{\mu _2\phi \mathscr {D}} \end{aligned}$$

where $g=9.81\hbox { m}/\hbox {s}^2$ is the acceleration due to gravity and $H_\mathrm{B}=10\,\hbox {cm}$. Other relevant properties include the brine viscosity, $\mu _{2}=1.090$ mPa s (Kestin et al. 1981), and the average diffusion coefficient in the bulk solution, $\mathscr {D}=1\times 10^{-9}\,\hbox {m}^2$/s. The latter is assumed to be independent of the solution concentration, based on observations reported in the literature for ethylene-glycol (EG)-water mixtures, where $\mathscr {D}=1.2-0.75\times 10^{-9}\,\hbox {m}^2$/s for $w_\mathrm{EG}=0-0.5$ (Ternström et al. 1996).

**Table 1:** Characteristic metrics of the density curves that represent the three solution pairs used in this study, namely maximum density difference between the two solutions ($\Delta \rho _\mathrm{max}/\rho _2$, where $\rho _2=1.040$ g/mL is the initial density of the brine), weight fraction at maximum density ($w^\mathrm{max}$) and at neutral buoyancy $w^0$. The Rayleigh number, *Ra*, is calculated from Eq. 1

Solution	$\rho _1$ (g/mL)	$\Delta \rho _\mathrm{max}/\rho _2$ (%)	$w^\mathrm{max}$	$w^0$	*Ra*
MEG55/brine	1.018	0.4	0.41	0.68	2150
MEG57/brine	1.025	0.6	0.47	0.78	3230
MEG59/brine	1.032	0.9	0.50	0.88	4610

### Experimental Procedure and Imaging

The bowl is wet-packed using solution 2 (brine) for about 90% of its volume, corresponding to a height, $H_\mathrm{B}\approx 13\,\hbox {cm}$, and it is placed on the bed of the scanning instrument (Universal Systems HD-350 X-ray CT scanner). A dense slurry of solution 1 (MEG) and beads is prepared separately and poured in the bowl carefully ($H_\mathrm{B}\approx 2\,\hbox {cm}$), so as to minimise disturbances to the interface between the two fluids. The bowl is covered with a clear plastic film and the first CT scan is taken. The time needed to pour the MEG slurry and to complete the first scan always took < 2 min. The entire bowl is then scanned every $10{-}30$ min for up to 10 h and by collecting a total of about 20 CT scans. Because one scan takes approximately one minute to complete, the obtained 3D reconstructions can be considered as still-frames of the mixing process at specific times. For image acquisition, the following set of parameters was applied: field-of-view ($24\times 24)\,\hbox {cm}^2$; energy level of radiation 120 eV; tube current 150 mA. The scanner is operated in helical mode with the pitch set to 1, the index to 2, the number of revolutions to 70 and the total scanning length to 140 mm; this produces 71 2 mm-thick tomograms per complete scan with a voxel size in the ($x-z$) plane of ($0.4688\times 0.4688)\,\hbox {mm}^2$. For subsequent analysis, the latter are averaged over a $5\times 5$ rectangular grid to produce ($2.3\times 2.3\times 2)\,\hbox {mm}^3$ cubic voxels, thereby reducing the uncertainty of the CT reading to 48 HU (corresponding to an error of approximately 10 wt.% on the measured solute concentration at the voxel level). We note that the main advantages in operating the scanner in the helical rather than the ‘stop-and-go’ mode are that (i) scanning time is significantly reduced (1–2 min vs. 10 min) and (ii) shaking of the beadpack is minimised. The procedure above is repeated for experiments with the three different MEG solutions listed in Table 1 and for each solution two repeats have been completed. Parameters that are specific to each experiment are summarised in Table 2 and their estimation is explained in the following section.

**Table 2:** Summary of experiments conducted in this study. The parameters listed in the table have been estimated upon following the procedure described in Sect. 2.3. $M_1$ and $M_2$ are the mass of solution 1 (MEG) and 2 (brine) with estimated uncertainty, $\sigma _\mathrm{M}$; $V_\mathrm{T}$ and $V_\mathrm{B}$ are the volumes of the top and bottom sections of the bowl and $\widehat{w}_\mathrm{B}(t_\mathrm{f})$ is the mass fraction of solute in the bottom section of the bowl at the end of the experiment. For each experiment, $H_\mathrm{T}=2\,\hbox {cm}$ and $H_\mathrm{B}=13\,\hbox {cm}$

Solution	$M_1$ (g)	$M_2$ (g)	$\sigma _\mathrm{M}$ (g)	$V_\mathrm{T}$ (mL)	$V_\mathrm{B}$ (mL)	$\widehat{w}_\mathrm{B}(t_\mathrm{f})$
MEG55/brine	88.7	792.4	4.9	242.2	2116	0.112
	97.8	788.7	5.4	267.0	2107	0.124
MEG57/brine	79.4	808.2	5.3	215.2	2159	0.098
	72.4	815.3	6.8	196.1	2178	0.089
MEG59/brine	83.3	799.1	7.9	224.1	2134	0.104
	103.9	784.0	10.2	279.6	2094	0.133

### Image Processing

In the derivation of the relevant operating equations in terms of CT numbers, the following assumptions apply: (i) the porosity is constant and uniform, (ii) volume changes on mixing the two liquid solutions are negligible and (iii) the CT number varies linearly with the weight fraction of KI in solution. Assumption (i) is justified in view of the large size of the voxels [$V_\mathrm{vox}\approx 11\,\hbox {mm}^3$ corresponding to about 100 beads and to an edge-length/bead diameter of $\approx 5$, which corresponds to the REV size typically assumed for uniform beadpacks (Clausnitzer and Hopmans 1999)]. Assumption (ii) applies in this study, because any volume change resulting from the mixing between solutions 1 and 2 is very small when compared to the voxel size ($<0.3$%rel.). Because of the small density changes associated with mixing ($<3$%rel.), we can further assume that volume and mass fractions of solute are approximately equal (see also Fig. 2b). Assumption (iii) has been verified with an independent experiment and the results are reported in “Appendix A1”. Given these assumptions, the following equation is obtained where the CT number in a voxel *i* at given time *t*, $CT_i(t)$, is expressed as the linear combination of the CT numbers associated with the volume and mass fractions of each of its components:

2 $$\begin{aligned} CT_i(t) = \phi \left[ w_i(t)CT_1+(1-w_i(t))CT_2\right] + (1-\phi )CT_\mathrm{s} \end{aligned}$$

where $CT_1$ (MEG) and $CT_2$ (brine) are the CT numbers of the pure liquids, while $CT_s$ is the CT number of the glass beads. As explained in the following, the latter conveniently drops out of the equation upon subtracting scans acquired at different times, while the CT numbers of the pure fluids can be obtained from a calibration that accounts for the material balance in the system.

**Fig. 3:**
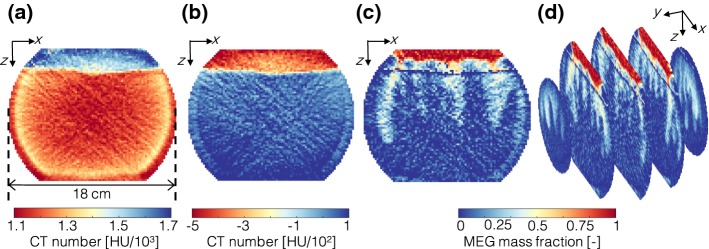
The adopted workflow for image processing. **a** The raw tomogram in terms of CT numbers (shown is the central slice of the bowl). **b** Reconstruction of the same slice obtained upon subtraction of scans acquired at different times (shown is the difference between final and initial scans); this procedure removes image noise and enables the identification of the initial interface between the two solutions. **c** Conversion of the tomogram to MEG fraction, $w_i(t)$, using Eq. 4. **d** Reconstruction of the entire bowl by applying the same methodology to each slice (total scanning length: 14 cm)

The workflow that has been followed for image processing is depicted in Fig. 3, where the central slice of the bowl is shown as an example of general validity. The raw tomogram is presented in Fig. 3a and evidences the presence of significant beam hardening around the periphery of the bowl. Subtraction of tomograms acquired at identical positions can effectively remove this effect, as shown in Fig. 3b. Here, the first ($t=t_0$) and final ($t=t_\mathrm{f}$) scans have been subtracted, further enabling the identification of the original interface between the two solutions. Accordingly, the volumes occupied initially by solutions 1 ($V_\mathrm{T}$, MEG) and 2 ($V_\mathrm{B}$, brine) are obtained upon application of a threshold value ($CT=-190$ HU in this study) and by counting the number of voxels *N* in each section, i.e. $V_j=N_jV_\mathrm{vox}$, where $j=T,B$. The corresponding total mass of solution 1 and 2 can be readily computed as $M_1= \phi V_\mathrm{T}\rho _1$ and $M_2= \phi V_\mathrm{B}\rho _2$. At the end of the experiment ($t=t_\mathrm{f}$), solution 1 (MEG) has completely dissolved and the top section of the bowl ($V_\mathrm{T}$) contains only solution 2 (brine, $\widehat{w}_\mathrm{T}(t_\mathrm{f})=0$); the corresponding value in $V_\mathrm{B}$ is obtained from the following material balance,

3 $$\begin{aligned} \widehat{w}_\mathrm{B}(t_\mathrm{f})=\frac{M_1}{M_1+M_2-\rho _2V_\mathrm{T}} \end{aligned}$$

We note that the attainment of an inverted concentration profile (as opposed to a uniform distribution) is expected in view of the large difference between the time-scale of convective fluxes and the diffusive counterpart ($\mathrm {Ra}>1000$), and the short duration of our experiments ($\mathcal {O}(t)\sim 100-1000$ min) relative to the time scale for diffusion ($\mathcal {O}(t)\sim 10^4-10^5$ min). The mass fraction of solute (MEG) in each voxel *i* in the top, $w_{\mathrm {T},i}(t)$, and bottom sections of the bowl, $w_{\mathrm {B},i}(t)$, can therefore be computed as follows:

4a $$\begin{aligned} w_{\mathrm {B},i}(t)&=\widehat{w}_\mathrm{B}(t_\mathrm{f})\frac{{CT}_{\mathrm {B},i}(t)-{CT}_\mathrm{B,i}(t_\mathrm{0})}{\widehat{CT}_\mathrm{B}(t_\mathrm{f})-\widehat{CT}_\mathrm{B}(t_{0})}\end{aligned}$$

4b $$\begin{aligned} w_{\mathrm {T},i}(t)&=1-\frac{{CT}_{\mathrm {T},i}(t)-{CT}_\mathrm{T}(t_{0})}{\widehat{CT}_\mathrm{T}(t_\mathrm{f})-\widehat{CT}_\mathrm{T}(t_{0})} \end{aligned}$$

where ${CT}_{\mathrm {T},i}(t)$ and ${CT}_{\mathrm {B},i}(t)$ are the time-dependent CT values in each voxel *i*, while $\widehat{CT}_\mathrm{B}$ and $\widehat{CT}_\mathrm{T}$ represent the average of all voxel CT values in the bottom and top sections of the bowl at the initial and final time ($t_{0}$ and $t_\mathrm{f}$, respectively). The latter are associated with the CT numbers of the pure liquid solutions and are obtained for each experiment independently, e.g. for the top section in Eq. 2: $\widehat{CT}_\mathrm{T}(t_\mathrm{f})-\widehat{CT}_\mathrm{T}(t_\mathrm{0}) = \phi (CT_2-CT_1)$. Equations 4a and 4b are applied on a voxel scale (as shown in Fig. 3c for the central slice of the bowl) and the operation is repeated for each slice in the bowl to enable the three-dimensional reconstruction of the temporal and spatial evolution of the process of convective mixing (Fig. 3d). As an important component in the analysis that follows, the temporal evolution of the total mass of solute in each section (top, *T*, and bottom, *B*) is estimated as:

5a $$\begin{aligned} m_{\mathrm {B}}(t)&=\rho (w_\mathrm{B})V_\mathrm{B} \end{aligned}$$

5b $$\begin{aligned} m_{\mathrm {T}}(t)&=\rho (w_\mathrm{T})V_\mathrm{T} \end{aligned}$$

where the density of the mixture is computed from the parameterisation of the curves shown in Fig. 2 as a function of the average mass fraction of the solute in the given section of the bowl, $w_\mathrm{B}(t)$ or $w_\mathrm{T}(t)$, which are estimated by using section-averaged *CT*(*t*) numbers in Eqs. 4a and 4b.

## Modelling

The outcomes from experiments presented in this study are compared to those associated with a purely diffusive scenario, so as to quantify any enhancement of mixing introduced by the convective dissolution process. In this study, this scenario is described by the numerical solution of the one-dimensional diffusion equation in a sphere, so as to closely represent the geometry used in the experiments. The equation can be written as:

6 $$\begin{aligned} A(z)\phi \frac{\partial c}{\partial t} = \frac{\partial }{\partial z}\left( \phi \mathscr {D}A(z)\frac{\partial c}{\partial z}\right) \end{aligned}$$

where *c* is the concentration of MEG in the brine solution, $\phi$ is the porosity, $\mathscr {D}$ is the molecular diffusion coefficient, and *z* and *t* are the spatial (vertical) and temporal coordinates. The cross-sectional area can be conveniently described as a function of *z*, i.e. $A(z) = \pi (z+h)[d-(z+h)]$ for $-h\le z\le d-h$, where *d* is the diameter of the sphere, *h* is defined so that $A(z=0)=\pi {d_\mathrm{t}}^2/4$ and *z* increases downwards (see Fig. 1). Equation 6 can be simplified further to give,

7 $$\begin{aligned} \frac{\partial c}{\partial t} =\mathscr {D}\left[ \frac{\partial ^2c}{\partial z^2} + \frac{\partial c}{\partial z}\left( \frac{1}{z+h}-\frac{1}{d-(z+h)}\right) \right] \end{aligned}$$

This partial differential equation is discretised in space using the finite-difference method with 500 grid points corresponding to a constant width $\Delta z\approx 0.3\,\hbox {mm}$. To this aim, the space derivatives are approximated using the central difference operator for each internal node and a no-flux condition is imposed at each boundary, i.e.

8 $$\begin{aligned} \left. \frac{\partial c}{\partial z}\right| _{z=z_\mathrm{t}}=\left. \frac{\partial c}{\partial z}\right| _{z=z_\mathrm{b}}=0 \end{aligned}$$

where $z_\mathrm{t}=0$ and $z_\mathrm{b}$ correspond to the top and bottom boundaries of the bowl (with diameter $d_\mathrm{t}$ and $d_\mathrm{b}$, as shown in Fig. 1). The system of 500 ordinary differential equations is solved in time using the ode15s solver in MATLAB with relative and absolute error tolerances set to 0.01% and $1\times 10^{-4}$ g/mL. As shown in Fig. 1, the following initial condition applies:

9 $$\begin{aligned} \mathrm {For}~t= & {} 0~\mathrm {and}~z_\mathrm{t}\le z\le H_\mathrm{T}: c = c_0=M_1/(\phi V_\mathrm{T}) \nonumber \\ \mathrm {For}~t= & {} 0~\mathrm {and}~H_\mathrm{T}< z\le z_\mathrm{b}: c = 0 \end{aligned}$$

where $H_\mathrm{T}$ and $V_\mathrm{T}$ are the thickness and volume of the initial MEG layer, and $M_1$ is the total mass of MEG (see Table 2). The mass of MEG in the top and bottom section of the bowl is computed as follows:

$$\begin{aligned} m_j(t)= \phi \displaystyle \int _{z_\mathrm{1}}^{z_2}c(t,z)A(z)dz \end{aligned}$$

where for $j=T$ (top), $z_1=z_\mathrm{t}$ and $z_2=H_\mathrm{T}$, while for $j=B$ (bottom), $z_1=H_\mathrm{T}+\Delta z$ and $z_2=z_\mathrm{b}$.

## Results

### Extent of Dissolution and Mixing Regimes

**Fig. 4:**
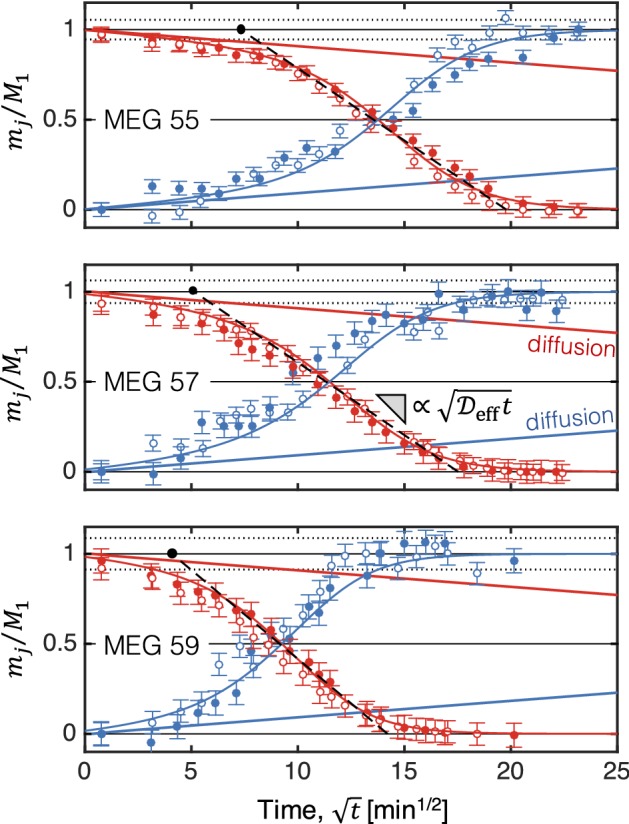
Relative mass of MEG dissolved in brine, $m_j/M_1$, as a function of the square root of time, $\sqrt{t}$, for experiments conducted with MEG55 (top), MEG57 (centre) and MEG59 (bottom). Two independent sets of experiments are shown for each scenario (filled and empty symbols). Colours refer to observations on the top (red) and bottom (blue) sections of the bowl. In each plot, the two sets of solid curves represent a purely diffusive scenario (straight lines, Eq. 7) and modified logistic functions fitted to the experimental data (equations and parameters given in “Appendix A2”). The black dashed lines are linear fits applied to the time period where the process of convective mixing attains a pseudo-diffusive regime; the corresponding parameters ($\mathscr {D}_\mathrm{eff}$, $t_\mathrm{c}$ and $t_\mathrm{s}$ are summarised in Table 3)

Figure 4 shows the fraction of solute (MEG) dissolved in brine, $m_j/M_1$, as a function of the square root of time, $t^*=\sqrt{t}$, for the three MEG solutions. For each system, the dissolved amount has been calculated for both top ($j=T$, red symbols) and bottom ($j=B$, blue symbols) sections of the bowl independently, and results are reported for two repeated experiments (empty and filled symbols). Error bars that have been estimated from the variance of the computed total mass of MEG, $M_1(t) = m_\mathrm{B}(t) + m_\mathrm{T}(t)$, are also shown, and are reported as $\sigma _\mathrm{M}$ in Table 2. In each plot, two sets of curves are also shown that represent (i) modified logistic functions fitted to the experimental data (solid curves, see “Appendix A2”) and (ii) the purely diffusive scenario (straight solid lines). The latter are the numerical solutions of the model presented in Sect. 3

Overall, the experiments show good reproducibility and they all delineate a behaviour that is characterised by three dissolution regimes, namely (i) diffusive, (ii) convection-dominated and (iii) shutdown. These regimes are associated with a marked change in the slope of the fitted logistic function and, accordingly, in the rate of dissolution. In particular, at early times ($t^*<1-5\,\hbox {min}^{0.5}$) all the experiments approach the behaviour predicted by a purely diffusive scenario, where the dissolved mass grows in proportion to $\sqrt{\mathscr {D}t}$, with $\mathscr {D}$ being the bulk molecular diffusion coefficient. With the onset of convection, the rate of dissolution increases significantly; notably, a second (pseudo-)diffusive regime is observed, which is denoted in the figure by the black dashed lines with slope proportional to $\sqrt{\mathscr {D}_\mathrm{eff}t}$. The effective diffusion coefficient, $\mathscr {D}_\mathrm{eff}$, can be readily estimated from the squared ratio of the slopes of these two (linear) regimes (diffusive and pseudo-diffusive); the obtained values are summarised in Table 3. For the three systems investigated, the ratio of the effective to the molecular (bulk) diffusion coefficient, $\mathscr {D}_\mathrm{eff}/\mathscr {D}$, takes an average value of $73\pm 5$ (MEG55), $74\pm 7$ (MEG57) and $110\pm 15$ (MEG59), corresponding to an enhancement of the rate of dissolution of about two orders of magnitude.

**Table 3:** Macroscopic measures of convective mixing extracted from the experiments carried out in this study. Rayleigh number (*Ra*), effective diffusion coefficient achieved in the convective regime ($\mathscr {D_\mathrm{eff}}$), onset time of convection ($t_\mathrm{c}$) and time of convective shutdown ($t_\mathrm{s}$). The molecular (bulk) diffusion coefficient takes the value $\mathscr {D}=1\times 10^{-5}\,\hbox {cm}^2$/s. The parameters and their uncertainties have been obtained using standard relationships for weighted linear regression (Taylor 1997)

Solution	*Ra*	$\mathscr {D}_\mathrm{eff}/\mathscr {D}$	$t_\mathrm{c}$ (min)	$t_\mathrm{s}$ (min)
MEG55/brine	2150	$79\pm 7$	$50\pm 9$	$363\pm 38$
		$67\pm 7$	$57\pm 13$	$423\pm 54$
MEG57/brine	3230	$78\pm 9$	$32\pm 9$	$314\pm 46$
		$69\pm 11$	$21\pm 9$	$300\pm 58$
MEG59/brine	4610	$115\pm 22$	$15\pm 8$	$190\pm 44$
		$105\pm 20$	$19\pm 9$	$215\pm 49$

**Fig. 5:**
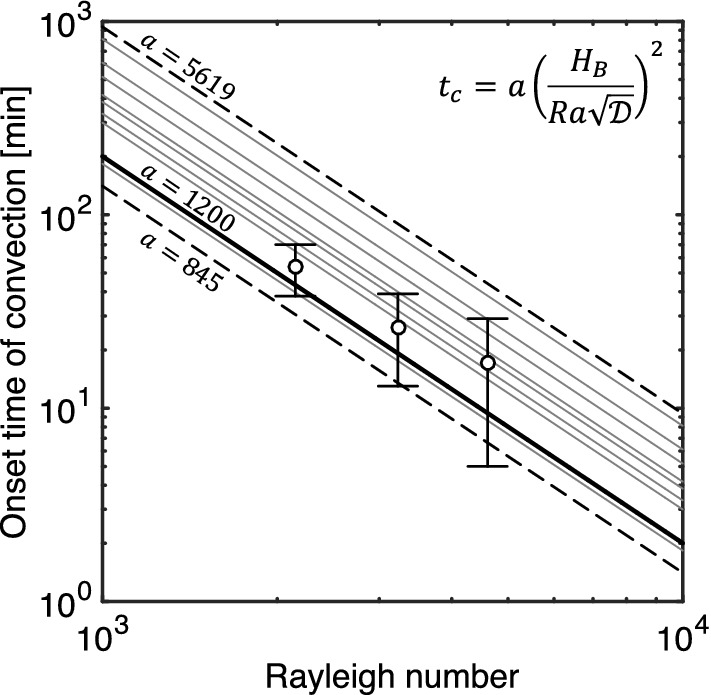
Onset time of convection as a function of the Rayleigh number for the three scenarios investigated in this study (symbols). The lines correspond to a correlation of the form $t_\mathrm{c}\sim \mathrm {Ra}^{-2}$ (equation given on the plot) that has been adopted in various numerical studies summarised in Emami-Meybodi et al. (2015) and that use different values of the prefactor *a*. Other parameters include the height of the domain, $H_\mathrm{B}=10\,\hbox {cm}$ and the molecular (bulk) diffusion coefficient, $\mathscr {D}=1\times 10^{-5}\,\hbox {cm}^2$/s

The experimental data confirm the expected positive trend in the onset and subsequent rate of dissolution with increasing Rayleigh number. The time for the onset of the convective regime, $t_\mathrm{c}$, has been estimated by identifying the point at which the experimental measurements depart from the model-predicted diffusive line. For each scenario, this point is denoted in Fig. 4 by the black circle, which has been obtained upon extrapolation of the trend predicted by the pseudo-diffusive regime back to $m_j/M_1=1$; the obtained values are $54\pm 16$ min (MEG55), $26\pm 13$ min (MEG57) and $17\pm 12$ min (MEG59), and are additionally plotted in Fig. 5 as a function of the Rayleigh number, *Ra*. It can be seen that our experimental observations compare favourably with results from numerical studies reported in the literature and summarised in Emami-Meybodi et al. (2015), where it is shown that $t_\mathrm{c}\sim \mathrm {Ra}^{-2}$. As it can be inferred from the figure, the determination of the onset of convection is affected by a significant degree of uncertainty (deviations of up to one order of magnitude are seen among the trends predicted by the numerical simulations). The latter results from the presence of perturbations at the interface, which need to be imposed artificially (in numerical simulations) or are naturally introduced by packing heterogeneities (as it is the case of our experiments). As shown in Fig. 4, the convective regime is followed by a gradual slow down of the dissolution rate that eventually approaches a value near zero. In our system, this shutdown appears because of the depletion of the MEG plume; accordingly, because of the trend in the rate of dissolution described above, the time to attain convective shutdown ($t_\mathrm{s}$ in Table 3) decreases with increasing *Ra* number, i.e. $t_\mathrm{s}=393\pm 66$ min (MEG55), $307\pm 74$ min (MEG57) and $202\pm 66$ min (MEG59).

Because it involves convection, the dissolution process is affected by hydrodynamic dispersion, the extent of which depends on the pore fluid velocity, $v=k\Delta \rho {g}/\mu _2\phi$. For the longitudinal dispersion coefficient, $D_\mathrm{L}\sim {Pe}$ (Perkins and Johnston 1963), with $Pe=vl/\mathscr {D}$ being the Péclet number and $l=d_\mathrm{P}$ the characteristic length scale. Transverse dispersion tends to diminish the amplitude of the concentration gradients in the system (Wang et al. 2016) and is therefore expected to slow down the dissolution process. It has also been reported the accounting for dispersion in numerical simulations can reduce the onset time of convection of up to two orders of magnitude (Hidalgo and Carrera 2009). On the one hand, there seems to be some general consensus that dispersion effects are small on both the pattern and time-scale of the density-driven dissolution process (Riaz et al. 2006; Slim and Ramakrishnan 2010; Chevalier et al. 2015). On the other hand, dispersion may be significant locally (Backhaus et al. 2011), where density differences are large ($\Delta \rho =\Delta \rho _\mathrm{max}$) and where $\mathcal {O}(Pe)\sim 10$ in our system. If any hydrodynamic dispersion is present in the experiments reported here, this is accounted for in the value of the estimated effective diffusion coefficient, $\mathscr {D_\mathrm{eff}}$, that lumps diffusive and dispersive processes together.

**Fig. 6:**
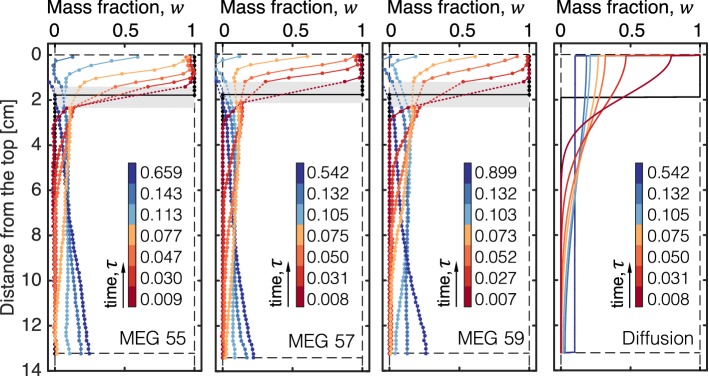
Horizontally averaged profiles of the MEG mass fraction, *w*, as a function of the distance from the top of the bowl, *z*. Results are shown for experiments carried out with the three MEG solutions (from left to right: MEG55, MEG57 and MEG59), while the rightmost panel shows predictions from a model describing the purely diffusive scenario described in Sect. 3. For each scenario, profiles are shown at different values of the dimensionless time, $\tau =\mathscr {D_\mathrm{eff}}t/H^2_\mathrm{B}$ ($\mathscr {D_\mathrm{eff}}=\mathscr {D}$ for pure diffusion), while the black solid line denotes the initial position of the interface. The grey-shaded area in the plots with experimental observations represents a region where image noise precludes a reliable estimate of the MEG mass fraction

### Horizontally Averaged Concentration Profiles

In Fig. 6, vertical profiles are presented of the mass fraction of solute, *w*, at various times and for the three systems investigated, namely MEG55, MEG57 and MEG59. The profiles have been computed upon using *CT* numbers in Eq. 4 that represent the average of all voxels in each 2 mm-thick horizontal section of the bowl. To facilitate comparison among observations with different MEG solutions (and, accordingly, *Ra* numbers), profiles are shown in the figure for CT scans that have been acquired at similar values of the dimensionless time, $\tau =\mathscr {D_\mathrm{eff}}t/H^2_\mathrm{B}\approx 0.01-0.6$. Results are also shown in the rightmost panel of the figure for the purely diffusive case and for which $\tau =\mathscr {D}t/H^2_\mathrm{B}$. In each plot, the black solid line represents the position of the interface at the start of the experiment. The experimental results obtained for different Rayleigh numbers show a significant degree of similarity in terms of both the temporal and spatial evolution of the dissolved plume: for $\tau <0.08$ (red profiles), the MEG/brine interface recedes gradually, while the solute plume moves downwards in the bowl, because of its larger density as compared to fresh brine; at $\tau \approx 0.1$, the pure MEG solution has almost completely dissolved and for $\tau >0.1$ the solute plume begins accumulating at the bottom of the bowl (blue profiles). Notably, this results in the reversal of the concentration gradient along the bowl, with the mass fraction of MEG in brine now increasing with the distance from the top. At the end of the experiment ($\tau \approx 0.7$), the mass fraction of MEG increases from $w\approx 0$ at $z=0\,\hbox {cm}$ to $w\approx 0.25$ at $z=13\,\hbox {cm}$. This late-time distribution of the solute differs from the corresponding profile predicted by the model that describes a purely diffusive scenario (rightmost panel), where -as expected- the solute reaches a uniform distribution along the entire length of the bowl. In other words, convection precludes a perfect dilution of the plume as it moves downwards and the resulting (stable) density gradient once convection ceases is such that diffusion remains the only mechanism to achieve complete mixing. Two additional observations arise from the comparison between the experiment and the diffusion model. First, because density is constant in the model and diffusion is ubiquitous, the MEG/brine interface does not show the characteristic receding behaviour observed in the experiments, where $\rho _1<\rho (w)$. In this context, despite being fully miscible with brine, the lower density of MEG acts towards stabilising the interface, while maintaining a much steeper concentration gradient across it. Second, prior to the cessation of convection the behaviour of the solute plume underneath the interface is similar to the one observed in the diffusion model, thus supporting the findings discussed above on the establishment of a pseudo-diffusive regime in the experiments.

### Three-Dimensional Imaging and Convective Patterns

**Fig. 7:**
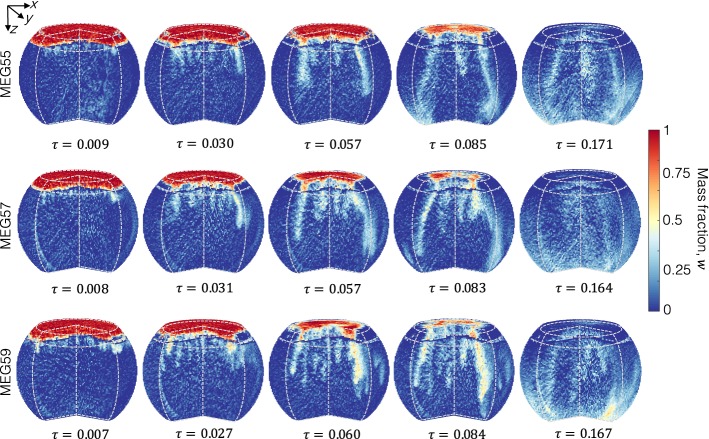
Three-dimensional reconstructions of the convective mixing process within the bowl, as obtained from X-ray CT scans. Images are shown in terms of solute (MEG) mass fraction, $w_i(t)$, for three systems, namely MEG55 (top row), MEG57 (middle row) and MEG59 (bottom row), as a function of the dimensionless time, $\tau =\mathscr {D}_\mathrm{eff}t/H^2_\mathrm{B}$. Voxel dimensions are: ($2.3\times 2.3\times 2)\,\hbox {mm}^3$

Figure 7 shows three-dimensional reconstructions of the bowl at various times for the experiments with MEG55 (top row), MEG57 (middle row) and MEG59 (bottom row). The solute mass fraction, *w*, has been calculated using Eq. 4 and the dimensionless time, $\tau$, is again chosen to facilitate the analysis and comparison of experiments conducted at different Rayleigh numbers ($\tau \approx 0.008-0.17$). In particular, the following regimes are identified from the 3D images: at early times ($\tau <0.01$, first column), a large number of small-scale finger projections ($\mathcal {O}(l)\sim 1\,\hbox {cm}$) are seen just underneath the MEG/brine interface; upon further dissolution ($0.01<\tau <0.1$, columns 2-4), the MEG layer continues to retract and the fingers continue to grow until they reach the bottom of the bowl ($\mathcal {O}(l)\sim 10\,\hbox {cm}$). Closer inspection of the images indicates that the mass fraction of MEG vary considerably among the different finger projections reaching values as high as $w=0.6-0.7$ in the centre of some of the fingers. By the time the MEG layer has completely dissolved ($\tau >0.15$, last column), the plume has reached the bottom of the bowl, where the solute accumulates. At this stage of the dissolution process, although a concentration gradient is still present, the associated density gradient is such that the system is stable and further mixing can be achieved only by diffusion (see also one-dimensional profiles shown in Fig. 6). We note that the regimes just described are observed in each experiment conducted in this study and their dynamics are very similar when the dimensionless time $\tau$ is considered. Accordingly, the 3D maps shown in Fig. 7 are strikingly similar in terms of the development and propagation of the fingers. These observations provide further support to the existence of a pseudo-diffusive regime throughout a large portion of the dissolution process with a characteristic time-scale $\tau =\mathscr {D}t/H^2_\mathrm{B}$. In agreement with previous studies on density-driven convection [e.g. Riaz et al. (2006)], we also observe that the number of fingers increases with *Ra*.

**Fig. 8:**
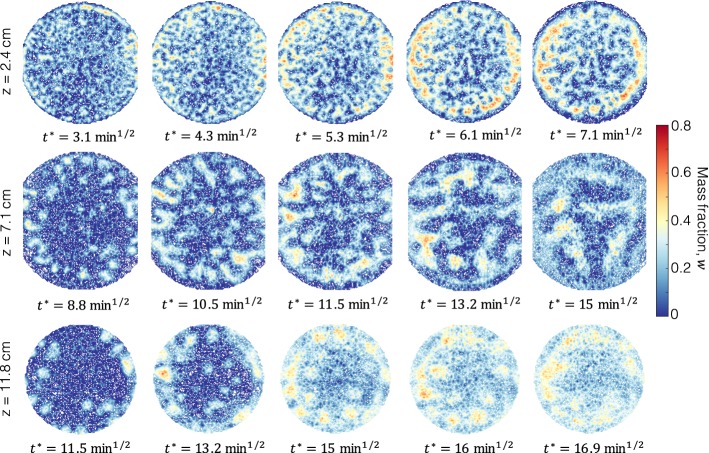
Two-dimensional horizontal flow patterns of convective mixing within the bowl for the experiment with MEG59. The horizontal cross sections represent three distinct positions within the bowl, namely $z=2.4\,\hbox {cm}$ (top row), $z=7.1\,\hbox {cm}$ (middle row) and $z=11.8\,\hbox {cm}$ (bottom row). In each row, time, $t^*=\sqrt{t}$, increases from left to right. Voxel dimensions are ($2.3\times 2.3\times 2)\,\hbox {mm}^3$ and the images are presented as contour lines of constant MEG mass fraction, $w_i(t)$

To discuss more in detail the temporal evolution of the characteristic spatial patterns that are formed throughout the dissolution process, Figure 8 shows 2D horizontal cross sections of the bowl at three vertical positions, namely $z=2.4\,\hbox {cm}$, $z=7.1\,\hbox {cm}$ and $z=11.8\,\hbox {cm}$ for the experiment conducted with MEG59 (in each row, time progresses from left to right). At early times and just underneath the interface ($z=2.4\,\hbox {cm}$, top row), many small protrusions are formed across the entire interface, most of which are still disconnected. With increasing time, the MEG concentration within the protrusions increases and bridges are formed, thus creating a structure that is largely connected. We note a strong similarity between these experimental observations and those reported in an earlier numerical study, where these connected structures have been described as a ‘maze’ (Fu et al. 2013). A similar behaviour is observed at a larger distance from the interface ($z=7.1\,\hbox {cm}$, middle row), although the numbers of fingers (at early times) and connected structures (at later times) is now significantly reduced. This decrease is due to the merging of fingers as they migrate downwards and create a coarser maze structure, where bridges of high solute concentration ($w\approx 0.2-0.4$) are separated by regions of near-zero concentration. Again, these structures are similar to those reported in one of the earliest investigations of convective mixing in three dimensions using MRI (Shattuck et al. 1995). The appearance at “early” times ($t\approx 130$ min) of islands of high concentration near the bottom of the bowl ($z=11.8\,\hbox {cm}$, bottom row) evidences that the columnar fingers can migrate downwards rather independently; notably, the cross-sectional area of these islands is considerably larger than their counterparts that originate higher up in the bowl due to the action of transverse dispersion during convection. This is also confirmed by the characteristic gradual discolouring of the islands that reflects the presence of an outward gradient in solute concentration. The overall increase in concentration across the entire cross section at later times is due to the accumulation of solute on the bottom of the bowl and the cessation of the convective process. It is worth pointing out that the emergence of the multidimensional structures just described is inherently not possible in 2D systems (e.g. Hele-Shaw cells) and it evidences the three-dimensional nature of the convective dissolution process. Because of the size of the system considered ($V\approx 2500\,\hbox {cm}^3$) and the high resolution of the images ($V_\mathrm{vox}\approx 0.010\,\hbox {cm}^3$), the observations presented here are thus first of its kind and demonstrate the ability of X-ray CT to provide quantitative information on the temporal and spatial evolution of the solute plume during density-driven convection in opaque porous media.

## Discussion

### Rate of Convective Dissolution and Mass Flux

**Fig. 9:**
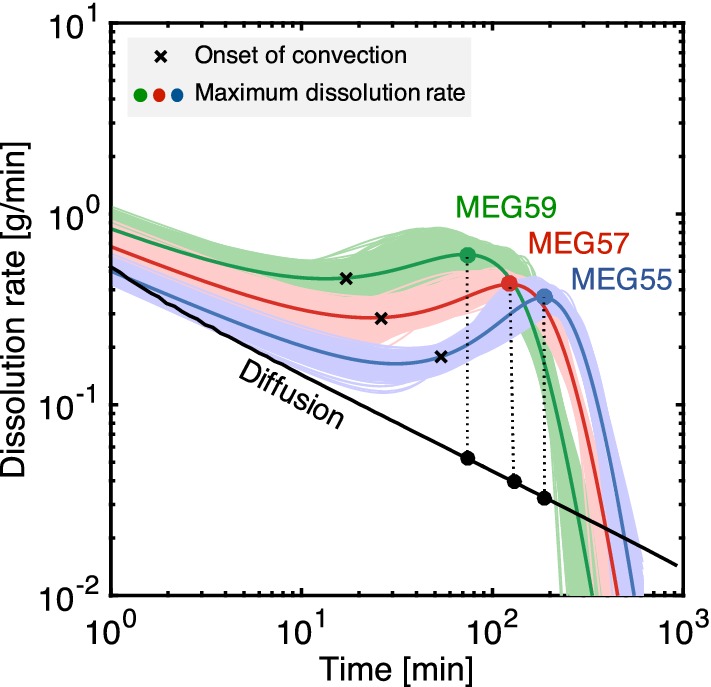
Rate of dissolution as a function of time for the experiments conducted with MEG55 (blue), MEG57 (red) and MEG(59) (green). The solid coloured lines are obtained upon differentiating the modified logistic function fitted to the experimental data (Fig. 4), while the solid black line is the numerical solution of the purely diffusive scenario (Sect. 3). For each MEG scenario, the colour-shaded region represents the ensemble of numerical realisations (300) conducted to account for the uncertainty of the raw experimental data. The cross-symbols are the rate of dissolution at the time of the onset of convection (estimated from Fig. 4), while the circles represent the time at which the maximum rate of dissolution is attained

The rate of dissolution is intuitively a key measure to quantify the enhancement of mixing (or lack thereof) produced by the convective process that originates from density instabilities in the system. Of particular interest is its comparison against the rate of dissolution that results from the action of diffusion and that relies solely on the presence of concentration gradients in the same system. This comparison is shown in Fig. 9, where the dissolution rate observed in the experiments conducted with the three MEG solutions is plotted as a function of time together with the rate predicted by the diffusion model described in Sect. 3. With reference to the results presented in Sect. 4.1 (Fig. 4), the dissolution rate is defined as,

10 $$\begin{aligned} r = -\frac{M_\mathrm{R}}{M_1}\frac{\hbox {d}m_\mathrm{T}}{\hbox {d}t} \end{aligned}$$

where $m_\mathrm{T}$ and $M_1$ are the current and the initial mass of MEG in the top section of the bowl, while $M_\mathrm{R}=100$ g is a reference mass used to re-establish dimensions and to enable comparison between the experiments where a different amount of MEG was used (see Table 2). The three solid curves obtained for the MEG solutions have been obtained by differentiating the logistic functions fitted to the experimental data. To account for the uncertainty of the experimental observations, 300 additional realisations of the fitting-differentiation exercise have been carried out by randomly varying the experimental data within the error bars shown in Fig. 4; these additional curves are also shown in the figure and create the colour-shaded regions around the mean curve of each system. It can be seen that all curves initially follow the trend predicted by the diffusion model (black solid line) and that they gradually diverge from it as time increases. In particular, the dissolution rate increases and reaches a maximum before falling off rapidly at late times. We acknowledge that this behaviour may not be ascribed solely to the effect of varying *Ra*, because of the effects introduced by the characteristic shape of the $\rho (w)$ curve of the three fluid pairs (Jafari Raad et al. 2016). Nevertheless, the observed trend closely reflects the attainment of the three regimes discussed in Sect. 4, namely diffusive, convection-dominated (or pseudo-diffusive, with onset-time $t_\mathrm{c}$ shown by the crosses) and shutdown. As expected, with increasing Rayleigh number the experimental curves depart sooner from the diffusive regime and they also reach a larger (and earlier) maximum dissolution rate, $r_\mathrm{max}$. For the three MEG systems, the obtained estimates are $r_\mathrm{max}=0.37\pm 0.06$ g/min (MEG55, at 186 min), $r_\mathrm{max}=0.43\pm 0.06$ g/min (MEG57 at 124 min) and $r_\mathrm{max}=0.61\pm 0.11$ g/min (MEG59 at 74 min). Interestingly, in all scenarios the time to reach maximum dissolution rates is about four times larger than the time required for the onset of convection, i.e. $t(r_\mathrm{max})\approx 4t_\mathrm{c}$. The black circles in Fig. 9 represent the rates of dissolution achieved by diffusion at equivalent (absolute) time and take values $r_\mathscr {D} = 0.033$ g/min (MEG55), $r_\mathscr {D} = 0.040$ g/min (MEG57) and $r_\mathscr {D} = 0.052$ g/min (MEG59), respectively. These dissolution rates are approximately one order of magnitude smaller than the corresponding values achieved in the presence of convection. We also note that by using a fixed boundary (i.e. the original interface) in our calculations the amount of MEG dissolved over time is underestimated. Accordingly, one should refer to a rate of MEG removal, rather than dissolution. This rate of removal combines two contributions: the rate of change in mass of buoyant solution (which is, effectively, the rate of dissolution) and the rate of change in the mass of non-buoyant solution ($w<w_0$). In our experiments, the latter is expected to be significantly smaller than the former, because, while some dissolved MEG does accumulate (temporarily) above the initial interface, a given amount also leaves the volume by convection. This last process is quite fast and effectively minimises the accumulation of solute above the interface. Accordingly, in our experiments the rate of MEG removal approaches the rate of dissolution. The latter is estimated with an uncertainty in the order of 15–20%, which we consider to be larger than any error introduced by using a fixed boundary in the calculations.

The Sherwood number, *Sh*, represents a non-dimensional measure of the convective mass flux and can be estimated from the ratio of the maximum convective dissolution rate computed above to the corresponding value in the presence of diffusion alone, while accounting for the appropriate length scales, i.e.

11 $$\begin{aligned} Sh =\frac{l_\mathrm{H}}{l_\mathscr {D}}\frac{(\hbox {d}m_\mathrm{T}/\hbox {d}t)_{H}}{(\hbox {d}m_\mathrm{T}/\hbox {d}t)_\mathscr {D}} \end{aligned}$$

where $l_\mathrm{H}=10~\mathrm {cm}\approx H_\mathrm{B}$ is the characteristic length scale of convective mixing, while $l_\mathscr {D}$ is the corresponding value associated with the diffusive process. In this study, the latter has been chosen to be the thickness of the diffusive boundary layer at the given time (threshold set to 5% deviation from the baseline) and take the value $l_\mathscr {D}\approx 1-2\,\hbox {cm}$ depending on the system considered. The corresponding estimates of the Sherwood number are therefore $Sh=57\pm 10$ (MEG55), $Sh=66\pm 9$ (MEG57) and $Sh=96\pm 17$ (MEG59). As discussed below, these estimates are in close agreement with the corresponding values obtained from the ratio of the effective-to-molecular diffusion coefficients presented in Table 3. We also note that we do not observe in Fig. 9 a clear regime of constant dissolution rate, in agreement with other experimental observations (Slim et al. 2013). The reasons for this are twofold; first, results from numerical simulations Slim (2014) suggest that for the range of Rayleigh number considered in this study ($\mathrm {Ra}=2150{-}4610$) the constant-flux regime is expected to be relatively short. Secondly, the chosen boundary condition in our experiments (constant volume of solute as opposed to a constant concentration of solute adopted in most numerical studies) is such that the limited amount of MEG solution precludes the attainment of a regime with constant dissolution rate prior to the depletion of the MEG layer. In this context, we note that both boundary and geometrical constraints, which are inherent to geologic settings, will play an important role in controlling the dissolution process and the degree of mixing that can be achieved.

### Sherwood–Rayleigh scaling and geological $\hbox {CO}_2$ storage

**Fig. 10:**
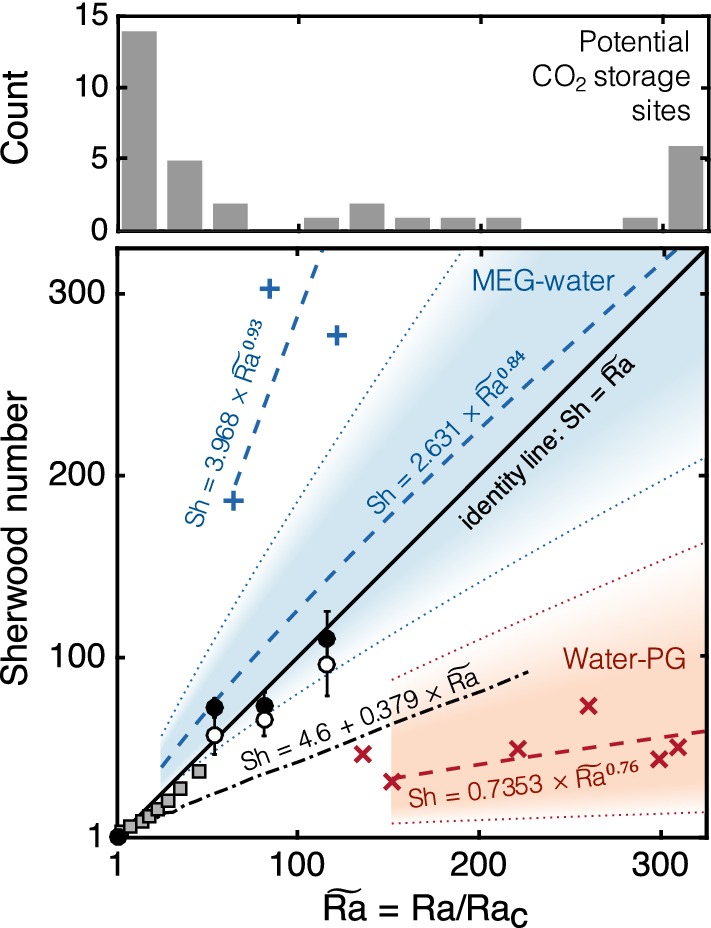
Convective mass flux plotted in terms of Sherwood number, *Sh* as a function of the Rayleigh number, *Ra*. Results from this study are reported as two sets of data that differ in the way *Sh* was calculated, namely as the ratio of effective-to-molecular diffusion coefficients (black filled circles, Table 3) or as the scaled ratio of the maximum dissolution rates (empty symbols, Eq. 11). Data from the literature include measurements using the MEG/brine system on 3D packings ($+$) (Wang et al. 2016) and with water/PG in a Hele-Shaw cell ($\times$) (Backhaus et al. 2011; Tsai et al. 2013). $Sh{-}Ra$ correlations reported in those studies are plotted as dashed lines (equations given in the figure) and the colour-shaded regions represent the uncertainties in the given parameters (Neufeld et al. 2010; Backhaus et al. 2011). Observations from thermal convection in three-dimensional porous media are also plotted and include results from both experiments [squares, Lister (1990)] and numerical simulations (dash-dot line, Hewitt et al. (2014)). The bar chart represents the sorting of 38 aquifers around the world according to the expected Rayleigh number (details provided in the manuscript text)

Observations of density-driven convection are often represented in the form of *Sh* vs. *Ra* plots aimed at identifying scaling laws that can be used to relate laboratory observations to field settings. The use of these dimensionless numbers is also needed as a means to compare observations from laboratory studies using different fluid pairs and geometries (e.g. 2D vs. 3D). In Fig. 10, we attempt this comparison by presenting the results from this study (circles) together with a selection of data and correlations found in the literature (details given in the figure caption). In the figure, the Rayleigh number has been normalised by its critical value, $Ra_\mathrm{c}$, defined as the *Ra* value for which *Sh* (or *Nu* in heat transfer studies) departs from the value 1 (Nield and Bejan 2006). It has been shown by numerous experimental studies that for convective flow to occur in a porous medium, $Ra>Ra_\mathrm{c}=4\pi ^2\approx 40$ (Katto and Masuoka 1967). We also purposely focus here on the range $\widetilde{Ra}=Ra/Ra_\mathrm{c}=1{-}300$ ($Ra=40{-}12000$), as this is the regime that is more likely to be expected at depth in potential geologic carbon sequestration sites (Sathaye et al. 2014), should the process of convective dissolution occur. We provide further support to this last observation with the bar chart also shown in Fig. 10 where data from 38 aquifers around the world are sorted according to the expected Rayleigh number. These include 11 major saline aquifers in the USA [$\widetilde{Ra}\sim 1{-}100$, 21 reservoirs in total compiled in Szulczewski et al. (2012)], 13 injection sites in the Alberta Basin ($\widetilde{Ra}\sim 1{-}10$) (Hassanzadeh et al. 2007) and the Sleipner site in the North Sea [$\widetilde{Ra}\sim 100{-}1000$, 4 cases depending on the assumed pressure/temperature conditions (Lindeberg and Wessel-Berg 1997)]. While these estimates must be used with some precaution due to the intrinsic difficulty in estimating suitable mean permeabilities and dimensions in heterogeneous reservoirs, the perception is that the condition $\widetilde{Ra}<100$ (${Ra}<4000$) may be typical in geologic reservoirs.

The data plotted in Fig. 10 evidence two aspects. First, the available data set is still quite scarce, particularly for $\mathcal {O}(Ra)\sim 1000$. A significant body of the literature exist on observations at low Ra values (${Ra}<1000$, corresponding to $\widetilde{Ra}<25$ in Fig. 10), including early studies on thermal convection in porous media [see a collection of more than 100 data points in Xie et al. (2012)] and more recent ones on dissolution-driven convection (Slim et al. 2013; Agartan et al. 2015). Others have focused on the high Rayleigh number regime ($\mathcal {O}(Ra)\sim 10^4{-}10^6$) (Neufeld et al. 2010; Kneafsey and Pruess 2010; Backhaus et al. 2011; Tsai et al. 2013; Ching et al. 2017; Nakanishi et al. 2016) and their observations fall outside the bounds of Fig. 10. Second, there is a significant degree of scatter among the reported results, which may be due to the use of 2D vs. 3D geometries, as well as of different model fluids. As discussed in the following, both aspects contribute to additional uncertainty on the fundamental behaviour of the dissolution flux and its dependence on the system parameters, such as the Rayleigh number.

The experiments carried out in the present study (circles) are well within the range expected in potential $\hbox {CO}_2$ storage sites lie near the identity line, suggesting that in this regime the dissolution flux increases linearly with Ra as $Sh=\alpha Ra$ with $\alpha \approx 1$. However, they disagree considerably with results reported on a supposedly similar experimental system, i.e. MEG/brine in a packed bed imaged by X-ray CT, for which a significantly larger dissolution has been reported ($\alpha \approx 4$, blue crosses in the figure) (Wang et al. 2016). We attribute this differences to the distinct shape of the density-concentration curve, in particular with the position of the maximum and cross-over points ($w^\mathrm{max}$ and $w^0$ in Fig. 2), which in Wang et al. (2016) are shifted towards larger MEG concentration values ($w^\mathrm{max}\approx 0.6$ and $w^0>0.9$). This further implies that the range of concentration values over which the MEG solution is no longer buoyant is wider and mixing rate is thus enhanced. This pattern has been quantitatively demonstrated by means of numerical simulations (Jafari Raad et al. 2016). It may not be surprising therefore that experimental data acquired on a different model fluid pair (Backhaus et al. 2011; Tsai et al. 2013), namely propylene glycol (PG) and water (red crosses in the figure), lie on the opposite corner of the diagram and suggest that the dissolution flux is significantly smaller (3–4 times when compared to our data at $\widetilde{Ra}=115$). In fact, for PG-water mixture the maximum and cross-over points of the density curve are shifted towards much *lower* values ($w^\mathrm{max}\approx 0.25$ and $w^0\approx 0.5$) when compared to the systems above (Dow Chemical 2017) and the mixing rate is thus expected to be significantly smaller (Hidalgo et al. 2012). For the PG-water system, the Sh-Ra correlation was also found to be nonlinear ($\mathrm {Sh}\sim Ra^{0.76}$, red dashed-line) with parameters affected by a relatively large uncertainty (as represented by the red shaded region in the figure). Interestingly, our results seem to follow more closely the correlation found in another study that used the MEG/brine system with similar density curves (Neufeld et al. 2010), although also in this case the scaling of the flux was found to be nonlinear (${Sh}\sim Ra^{0.84}$, blue dashed-line) and the uncertainty on the obtained parameters is admittedly large (represented by the blue shaded region in the figure).

As anticipated above, one of the key observations from the results obtained in study is the attainment of a linear $Sh\sim Ra$ scaling. Interestingly, this behaviour has been observed in studies on thermal convection in porous media, including observations from experiments (Xie et al. 2012) and numerical simulations in both two- (Hewitt et al. 2013) and three dimensions (Hewitt et al. 2014). The latter are shown in the plot with the dash-dotted line and predict a flux that is approximately three times smaller than the values observed in this study ($\alpha =0.379$). We note that the linear scaling is specific to Rayleigh numbers that are relatively small ($Ra_\mathrm{c}<Ra<\mathcal {O}(Ra)\sim 10^3$), while *Sh* is expected to become independent of *Ra* for $Ra>\mathcal {O}(Ra)\sim 10^4$ (Hidalgo et al. 2012; Slim 2014; Ching et al. 2017). Most significantly, our data seem to extend the results from one of the (very) few experimental studies reported in the literature where density-driven convection was investigated in a three-dimensional porous medium (grey-shaded square symbols) (Lister 1990). More observations within this important regime of Rayleigh numbers are needed to corroborate these findings, because at this stage we cannot exclude a priori that our data are affected by the characteristic density behaviour of aqueous MEG solutions (Jafari Raad et al. 2016). Nevertheless, the conclusion can be drawn that in the regime $1<\widetilde{Ra}<100$ ($40<Ra<4000$) and irrespectively of the chosen model fluid (pair), the dissolution flux increases linearly with *Ra* reaching values that are $40-100$ times larger than predictions based on diffusion alone. In the context of geological $\hbox {CO}_2$ storage, this could result in a reduction in the time scale for dissolution from $\sim 80{,}000$ years down to $\sim 1500$ years in a 50 m-thick permeable aquifer.

## Concluding Remarks

We have presented an experimental study on dissolution-driven convection imaged by X-ray CT in a uniform porous medium with MEG-water as model fluid pair. We obtain very good experimental reproducibility in terms of macroscopic measures of mixing, such as onset time of convection, maximum dissolution rate and averaged concentration profiles. Together with the recent work by Nakanishi et al. (2016) and Wang et al. (2016), we provide what are, to our knowledge, the first non-invasive determinations of three-dimensional patterns in opaque, random porous media in the regime $\mathcal {O}(Ra)\sim 1000$. The tomograms reveal the emergence and evolution of characteristic concentration structures, which are imaged at a resolution of 10 $\hbox {mm}^3$ from the onset of convection until its shutdown. The experimental observations are compared to the limiting numerical case of a purely diffusive scenario and are well described by a relationship of the form $Sh=0.025Ra$ for $Ra<5000$.

In agreement with previous findings, the comparison with results from other experimental studies suggests that the extrapolation of observations on analogue model fluids to the $\hbox {CO}_2$/brine system should be done with caution, due to effects introduced by the characteristic shape of the density-concentration curve. We contend that similar risks are posed by the use of simplified two-dimensional systems to mimic a porous medium and to model a process that is inherently three-dimensional. We also observe that there is a lack of direct experimental observations in the regime $\mathcal {O}(Ra)\sim 100{-}1000$, where subsurface processes are very likely to operate. We demonstrate that X-ray CT allows for precise imaging of solute concentrations at a resolution of about ($2\times 2\times 2)\,\hbox {mm}^3$, thus providing highly resolved spatial and temporal information on the fundamental behaviour of the convective process. This novel ability is key towards providing more realistic estimates on the extent of dissolution-driven convection in natural environments, because their inherent heterogeneity is likely to play a fundamentally important role in the determination of the convective flow pattern.

## Figures and Tables

**Fig. 11:**
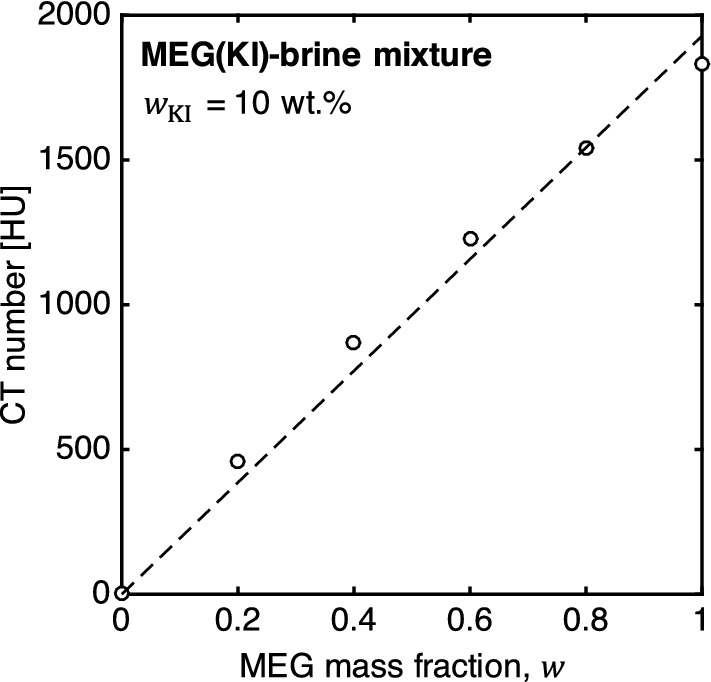
CT number as a function of the mass fraction of MEG in water. The MEG solution contains 10 wt% KI that is used as dopant in the experiments of convective mixing. The dashed line is a linear fit to the experimental values ($R^2=0.996$)

## Appendix A1: X-ray Imaging of Aqueous MEG Solutions and Density Curves

In this study, potassium iodide (KI) was used as dopant in the MEG solution ($\sim$ 9 wt%) to enable precise imaging of the solute plume. In the derivation of the operating equation used to compute the solute concentration from the X-ray tomograms (Sect. 2.3) it was assumed that the CT number varies linearly with the weight fraction of KI in solution. This assumption is verified against independent measurements of the measured CT number of solutions of MEG (10wt.% KI) mixed with water, as shown in Fig. 11. The following imaging parameters were applied: field-of-view ($24\times 24)\,\hbox {cm}^2$; energy level of radiation 120 eV; tube current 150 mA.

Throughout the experiment, the CT readings are converted into mass fraction of MEG in solution using Eq. 4. The density in each voxel is then computed from the parameterisation of the curves shown in Fig. 2 as a function of the average mass fraction of the solute. The curves have been fitted using polynomials of the form, $\rho =a_0+a_1w+a_2w^2+a_3w^3$, with parameters given in Table 4 and whose values were found by minimising the squared error, *E*:

A-1 $$\begin{aligned} E=\sum _{i=1}^{N} \left( \rho _i^\mathrm{exp}-\rho _i^\mathrm{mod}\right) ^2 \end{aligned}$$

where *N* is the number of experimental points, while $\rho ^\mathrm{exp}$ and $\rho ^\mathrm{mod}$ are the experimental and calculated density values. The values of *E* are also given in Table 4.

**Table 4:** Parameters of the fitted density curves shown in Fig. 2 with functional form, $\rho =a_0+a_1w+a_2w^2+a_3w^3$, together with the corresponding values of the squared error, *E* (Eq. A-1)

Solution	$a_0$ (g/mL)	$a_1$ (g/mL)	$a_2$ (g/mL)	$a_3$ (g/mL)	*E* ((g/mL)$^2$)
MEG55/brine	1.04	0.0131	0.0151	$-$ 0.0503	$7.0\times 10^{-7}$
MEG57/brine	1.04	0.0179	0.0136	$-$ 0.0466	$4.1\times 10^{-7}$
MEG59/brine	1.04	0.0306	$-$ 0.0086	$-$ 0.0300	$6.5\times 10^{-7}$

## Appendix A2: Modified Logistic Function

A modified logistic function was used to describe the temporal evolution of the dissolved mass, $m_j(t)$, in the bottom ($j=B$) and top ($j=T$) sections of the bowl. In particular, the logistic function has been modified so as to attain the correct limiting behaviour at early times, i.e.

A-2 $$\begin{aligned} \mathrm {For}~t\rightarrow 0: \frac{m_B(t)}{M_1}=Kt^* \end{aligned}$$

where $t*=\sqrt{t}$ and *K* is the slope of the straight line obtained from the numerical solution of the diffusion equation (see Sect. 3) when plotted as $m_B(t)/M_1$ vs. $\sqrt{t}$. In this study, $K=0.0096\,\hbox {min}^{-0.5}$. The modified logistic function reads therefore as follows:

A-3 $$\begin{aligned} \frac{m_B(t)}{M_1}=Kt^*+\frac{1-Kt^*}{\left[ 1+a_1e^{(-a_2(t^*-a_4))}\right] ^{1/a_3}} \end{aligned}$$

where $a_1$, $a_2$, $a_3$ and $a_4$ are fitting parameters obtained by matching Eq. A-3 to the experimental data and $m_T(t)=1-m_B(t)$. The values of the obtained parameters are summarised in Table 5, together with the value of the squared error, *E*:.

A-4 $$\begin{aligned} E=\frac{1}{M_1}\sum _{i=1}^{N} (m_{\mathrm {T}i}^\mathrm{exp}-m_{\mathrm {T}i}^\mathrm{mod})^2 \end{aligned}$$

where *N* is the number of experimental points, while $m_{\mathrm {T}}^\mathrm{exp}$ and $m_{\mathrm {T}}^\mathrm{mod}$ are the experimental and calculated mass values in the top section of the bowl.

**Table 5:** Parameters of the logistic function, Eq. A-3, fitted to measured relative amount of dissolved mass as a function of the square root of time, together with the corresponding values of the squared error, *E* (Eq. A-4). The experimental data are shown in Fig. 4 together with the corresponding fitted curves

Solution	$a_1$	$a_2$	$a_3$	$a_4$	*E*
MEG55/brine	1.4341	0.5617	1.4345	14.22	0.0037
	1.3972	0.4683	1.2949	15.15	0.0044
MEG57/brine	0.0461	0.5242	1.4778	19.33	0.0073
	1.3960	0.4915	1.5600	12.43	0.0057
MEG59/brine	1.1921	0.4984	1.0271	8.969	0.0037
	1.0925	0.6883	2.1626	11.75	0.0034

## References

[CR1] Agartan E, Trevisan L, Cihan A, Birkholzer J, Zhou Q, Illangasekare TH (2015). Experimental study on effects of geologic heterogeneity in enhancing dissolution trapping of supercritical $$\text{CO}_2$$. Water Resour. Res..

[CR2] Arendt B, Dittmar D, Eggers R (2004). Interaction of interfacial convection and mass transfer effects in the system $$\text{ CO }_2$$-water. Int. J. Heat Mass Transf..

[CR3] Backhaus S, Turitsyn K, Ecke RE (2011). Convective instability and mass transport of diffusion layers in a Hele-Shaw geometry. Phys. Rev. Lett..

[CR4] Benson SM, Cole DR (2008). $$\text{ CO }_2$$ sequestration in deep sedimentary formations. Elements.

[CR5] Bories S, Thirriot C (1969). Échanges thermiques et tourbillons dans une couche poreuse horizontale. La Houille Blanche.

[CR6] Chevalier S, Faisal TF, Bernabe Y, Juanes R, Sassi M (2015). Numerical sensitivity analysis of density driven $$\text{ CO }_2$$ convection with respect to different modeling and boundary conditions. Heat Mass Transf..

[CR7] Ching JH, Chen P, Tsai PA (2017). Convective mixing in homogeneous porous media flow. Phys. Rev. Fluids.

[CR8] Clausnitzer V, Hopmans J (1999). Determination of phase-volume fractions from tomographic measurements in two-phase systems. Adv. Water Resour..

[CR9] Diersch HJ, Kolditz O (2002). Variable-density flow and transport in porous media: approaches and challenges. Adv. Water Resour..

[CR10] Dow Chemical: Propylene glycols—density values (2017). https://dow-answer.custhelp.com/app/answers/detail/a_id/7471

[CR11] Ecke RE, Backhaus S (2016). Plume dynamics in Hele-Shaw porous media convection. Philos. Trans. R. Soc. A.

[CR12] Efika EC, Hoballah R, Li X, May EF, Nania M, Sanchez-Vicente Y, Trusler JM (2016). Saturated phase densities of ($$\text{ CO }_2+\text{ H }_2\text{ O }$$) at temperatures from (293 to 450) K and pressures up to 64 MPa. J. Chem. Thermodyn..

[CR13] Emami-Meybodi H, Hassanzadeh H, Green CP, Ennis-King J (2015). Convective dissolution of $$\text{ CO }_2$$ in saline aquifers: progress in modeling and experiments. Int. J. Greenh. Gas Control.

[CR14] Ennis-King JP, Paterson L (2005). Role of convective mixing in the long-term storage of carbon dioxide in deep saline formations. SPE J..

[CR15] Farajzadeh R, Zitha PL, Bruining J (2009). Enhanced mass transfer of $$\text{ CO }_2$$ into water: experiment and modeling. Ind. Eng. Chem. Res..

[CR16] Fu X, Cueto-Felgueroso L, Juanes R (2013). Pattern formation and coarsening dynamics in three-dimensional convective mixing in porous media. Philos. Trans. R. Soc. A.

[CR17] Gebhart B, Pera L (1971). The nature of vertical natural convection flows resulting from the combined buoyancy effects of thermal and mass diffusion. Int. J. Heat Mass Transf..

[CR18] Hassanzadeh H, Pooladi-Darvish M, Keith DW (2007). Scaling behavior of convective mixing, with application to geological storage of $$\text{ CO }_2$$. AIChE J..

[CR19] Hewitt DR, Neufeld JA, Lister JR (2013). Convective shutdown in a porous medium at high Rayleigh number. J. Fluid Mech..

[CR20] Hewitt DR, Neufeld JA, Lister JR (2014). High Rayleigh number convection in a three-dimensional porous medium. J. Fluid Mech..

[CR21] Hidalgo JJ, Carrera J (2009). Effect of dispersion on the onset of convection during $${CO}_2$$ sequestration. J. Fluid Mech..

[CR22] Hidalgo JJ, Fe J, Cueto-Felgueroso L, Juanes R (2012). Scaling of convective mixing in porous media. Phys. Rev. Lett..

[CR23] Howle L, Behringer R, Georgiadis J (1993). Visualization of convective fluid flow in a porous medium. Nature.

[CR24] Howle L, Behringer R, Georgiadis J (1997). Convection and flow in porous media. Part 2. Visualization by shadowgraph. J. Fluid Mech..

[CR25] Huppert HE, Neufeld J (2014). The fluid mechanics of carbon dioxide sequestration. Ann. Rev. Fluid Mech..

[CR26] Jafari Raad SM, EmamiMeybodi H, Hassanzadeh H (2016). On the choice of analogue fluids in $$\text{ CO }_2$$ convective dissolution experiments. Water Resour. Res..

[CR27] Katto Y, Masuoka T (1967). Criterion for the onset of convective flow in a fluid in a porous medium. Int. J. Heat Mass Transf..

[CR28] Kestin J, Khalifa HE, Correia RJ (1981). Tables of the dynamic and kinematic viscosity of aqueous NaCl solutions in the temperature range 20–150$$^\circ $$C and the pressure range 0.1–35 MPa. J. Phys. Chem. Ref. Data.

[CR29] Khosrokhavar R, Elsinga G, Farajzadeh R, Bruining H (2014). Visualization and investigation of natural convection flow of $$\text{ CO }_2$$ in aqueous and oleic systems. J. Pet. Sci. Eng..

[CR30] Kneafsey TJ, Pruess K (2010). Laboratory flow experiments for visualizing carbon dioxide-induced, density-driven brine convection. Transp. Porous Media.

[CR31] Knorr B, Xie Y, Stumpp C, Maloszewski P, Simmons CT (2016). Representativeness of 2D models to simulate 3D unstable variable density flow in porous media. J. Hydrol..

[CR32] Lindeberg E, Wessel-Berg D (1997). Vertical convection in an aquifer column under a gas cap of $$\text{ CO }_2$$. Energy Convers. Manag..

[CR33] Lister C (1990). An explanation for the multivalued heat transport found experimentally for convection in a porous medium. J. Fluid Mech..

[CR34] Macminn CW, Juanes R (2013). Buoyant currents arrested by convective dissolution. Geophys. Res. Lett..

[CR35] Moghaddam RN, Rostami B, Pourafshary P, Fallahzadeh Y (2012). Quantification of density-driven natural convection for dissolution mechanism in $$\text{ CO }_2$$ sequestration. Transp. Porous Media.

[CR36] Nakanishi Y, Hyodo A, Wang L, Suekane T (2016). Experimental study of 3D Rayleigh–Taylor convection between miscible fluids in a porous medium. Adv. Water Resour..

[CR37] Nazari Moghaddam R, Rostami B, Pourafshary P (2015). Scaling analysis of the convective mixing in porous media for geological storage of $$\text{ CO }_2$$: an experimental approach. Chem. Eng. Commun..

[CR38] Neufeld JA, Hesse MA, Riaz A, Hallworth MA, Tchelepi HA, Huppert HE (2010). Convective dissolution of carbon dioxide in saline aquifers. Geophys. Res. Lett..

[CR39] Newell DL, Carey JW, Backhaus SN, Lichtner P (2018). Experimental study of gravitational mixing of supercritical $$\text{ CO }_2$$. Int. J. Greenh. Gas Control.

[CR40] Nield DA, Bejan A (2006). Convection in Porous Media.

[CR41] Pau GSH, Bell JB, Pruess K, Almgren AS, Lijewski MJ, Zhang K (2010). High-resolution simulation and characterization of density-driven flow in $$\text{ CO }_2$$ storage in saline aquifers. Adv. Water Resour..

[CR42] Perkins TK, Johnston OC (1963). A review of diffusion and dispersion in porous media. SPE J..

[CR43] Raad SMJ, Hassanzadeh H (2015). Onset of dissolution-driven instabilities in fluids with nonmonotonic density profile. Phys. Rev. E.

[CR44] Riaz A, Hesse M, Tchelepi H, Orr F (2006). Onset of convection in a gravitationally unstable diffusive boundary layer in porous media. J. Fluid Mech..

[CR45] Sathaye KJ, Hesse MA, Cassidy M, Stockli DF (2014). Constraints on the magnitude and rate of $$\text{ CO }_2$$ dissolution at Bravo Dome natural gas field. Proc. Natl. Acad. Sci..

[CR46] Shattuck M, Behringer R, Johnson G, Georgiadis J (1995). Onset and stability of convection in porous media: visualization by magnetic resonance imaging. Phys. Rev. Lett..

[CR47] Slim AC (2014). Solutal-convection regimes in a two-dimensional porous medium. J. Fluid Mech..

[CR48] Slim AC, Ramakrishnan T (2010). Onset and cessation of time-dependent, dissolution-driven convection in porous media. Phys. Fluids.

[CR49] Slim AC, Bandi M, Miller JC, Mahadevan L (2013). Dissolution-driven convection in a Hele-Shaw cell. Phys. Fluids.

[CR50] Szulczewski ML, MacMinn CW, Herzog HJ, Juanes R (2012). Lifetime of carbon capture and storage as a climate-change mitigation technology. Proc. Natl. Acad. Sci..

[CR51] Taylor JR (1997). An Introduction to Error Analysis: The Study of Uncertainty in Physical Measurements.

[CR52] Ternström G, Sjöstrand A, Aly G, Jernqvist Å (1996). Mutual diffusion coefficients of water + ethylene glycol and water + glycerol mixtures. J. Chem. Eng. Data.

[CR53] Tsai PA, Riesing K, Stone HA (2013). Density-driven convection enhanced by an inclined boundary: implications for geological $$\text{ CO }_2$$ storage. Phys. Rev. E.

[CR54] Wang L, Nakanishi Y, Hyodo A, Suekane T (2016). Three-dimensional structure of natural convection in a porous medium: effect of dispersion on finger structure. Int. J. Greenh. Gas Control.

[CR55] Xie Y, Simmons CT, Werner AD, Diersch H (2012). Prediction and uncertainty of free convection phenomena in porous media. Water Resour. Res..

[CR56] Yang C, Gu Y (2006). Accelerated mass transfer of $$\text{ CO }_2$$ in reservoir brine due to density-driven natural convection at high pressures and elevated temperatures. Ind. Eng. Chem. Res..

